# Ophthalmic corticosteroids-related adverse events: the FDA adverse event reporting system (FAERS) database pharmacovigilance study

**DOI:** 10.3389/fphar.2024.1502047

**Published:** 2024-12-11

**Authors:** Chengzhi Liu, Xinyu Wang, Xusheng Cao

**Affiliations:** Beijing Tongren Eye Center, Beijing Ophthalmology and Visual Science Key Lab, Beijing Tongren Hospital, Capital Medical University, Beijing, China

**Keywords:** FAERS, corticosteroids, adverse event, pharmacovigilance, signal mining, ocular diseases

## Abstract

**Background:**

Corticosteroids are extensively used in ophthalmology, particularly for treating various inflammatory conditions. Despite their effectiveness, prolonged or high-dose corticosteroid use is associated with significant adverse drug reactions (ADRs), such as increased intraocular pressure, cataract formation, and secondary infections. However, there is currently no systematic study comparing the side effects of ophthalmic corticosteroids. This study aims to investigate the safety profiles of ophthalmic corticosteroids through pharmacovigilance analysis using the FDA Adverse Event Reporting System (FAERS) database.

**Methods:**

We conducted a retrospective analysis of ADR reports related to commonly used ophthalmic corticosteroids from the FAERS database, covering the period from Q1 2004 to Q4 2023. Clinical features such as gender, age, administration route, and dosage form were also analyzed. Signal detection methods, including Reporting Odds Ratio (ROR), Proportional Reporting Ratio (PRR), Bayesian Confidence Propagation Neural Network (BCPNN), and the Multi-Item Gamma Poisson Shrinker (MGPS), were used to identify potential safety signals.

**Results:**

A total of 9,854 ADRs related to ophthalmic corticosteroids were retrieved, with the most frequently reported drugs being Ozurdex (1,784 cases), Lotemax (3,239 cases), and Durezol (2,789 cases). Women accounted for a higher proportion of ADRs across most corticosteroids. ADR induction time analysis results showed that ADRs tend to occur in the early stages of drug use. The most common ophthalmic ADRs identified included eye inflammation, cataract, visual impairment, uveitis, eye pain, blurred vision, and retinal detachment. Additionally, Maxidex has been linked to endocrine disorders, while Ozurdex, Iluvien, and Triesence exhibited significant signals for product issues, likely related to their intraocular injection procedures. Notably, cataract was the most common PT among these drugs.

**Conclusion:**

Our study reveals significant safety concerns related to using ophthalmic corticosteroids, particularly regarding adverse reactions that can impact visual function. These findings highlight the need for careful monitoring and individualized treatment plans to minimize the risk of ADRs in patients receiving corticosteroid therapy. Future studies combining FAERS data with large-scale clinical research are needed to explore these safety concerns further.

## 1 Introduction

**FIGURE 1:**
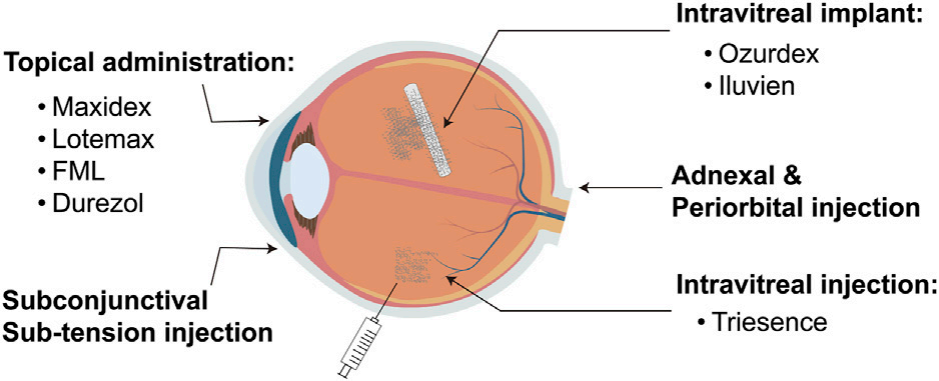
Routes of administration for ophthalmic corticosteroids.

Corticosteroids are extensively used in ophthalmology, particularly for treating various inflammatory conditions such as uveitis, optic neuritis, thyroid-associated ophthalmopathy, and other ophthalmic inflammatory diseases (Raizman, 1996; McGhee et al., 2002). They possess powerful anti-inflammatory and immunosuppressive properties, enabling them to rapidly control inflammation, relieve symptoms, and help preserve vision (Cutolo et al., 2019). Corticosteroids are available in various dosage forms and administration methods in ophthalmology, with a specific choice depending on the type and severity of the condition (Kiernan and Mieler, 2012; Puglia et al., 2021; Romano et al., 2023). Common forms include topical eye drops, eye ointments, intraocular injections, and systemic medications such as oral or intravenous injections (Figure 1). Topical eye drops and ointments are commonly used for anterior eye diseases like keratitis and anterior uveitis, delivering medication directly to the affected area. Intraocular injections are preferred for treating posterior eye conditions such as posterior uveitis and diabetic retinopathy, providing efficient local drug concentrations. Additionally, low-solubility Corticosteroid polymer sustained-release systems are employed for long-term intraocular treatments, such as in cases of diabetic macular edema, to ensure prolonged therapeutic effects (Busch et al., 2018). Systemic Corticosteroids are reserved for severe or widespread inflammatory diseases, including optic neuritis (Fung et al., 2020; Gaballa et al., 2021).

Although Corticosteroids are crucial in treating ophthalmic diseases, long-term or high-dose use can lead to side effects such as increased intraocular pressure, cataract formation, corneal ulcers, and secondary infections (Huscher et al., 2009; Roberti et al., 2020). Therefore, ophthalmologists must carefully balance the benefits and risks, tailoring individualized treatment plans based on the specific disease, its severity, and patient conditions (Bucolo et al., 2018). The choice of Corticosteroid dosage form and application can also vary depending on the disease’s severity and location and the individual patient’s circumstances (Caplan et al., 2017; Boscia et al., 2024). However, no current studies provide sufficient data to compare the safety profiles of different glucocorticoid drugs, highlighting the need for further research in this area.

The U.S. Food and Drug Administration Adverse Event Reporting System (FAERS) is a public, accessible, and free database containing millions of voluntarily submitted adverse drug reactions (ADRs) reports from healthcare professionals, consumers, and manufacturers (Hoffman et al., 2014). It is an important tool for post-marketing drug safety surveillance and signal detection and is widely used in pharmacovigilance research, including ocular ADRs (Fang et al., 2019; Ma et al., 2022; Zhao et al., 2023). Signal detection from FAERS data has become a recognized and effective method to identify potential safety risks associated with medications in real-world settings. By utilizing FAERS data, researchers can identify disproportionate reporting of ADRs, helping to establish hypotheses about causal relationships between drugs and adverse events, particularly for rare or severe outcomes (Fukazawa et al., 2018; Subeesh et al., 2018).

Despite the widespread use of glucocorticoids in ophthalmology, there is limited real-world data on their associated ADRs. Therefore, this study aims to utilize the FAERS database to conduct a pharmacovigilance analysis of glucocorticoids used in ophthalmology, identifying potential ADR signals and evaluating their severity and clinical impact. The findings from this research will contribute to a better understanding of the safety profile of glucocorticoids in ophthalmic settings, offering valuable insights for clinicians in minimizing the risk of adverse outcomes while optimizing therapeutic benefits.

## 2 Methods

### 2.1 Data source

This study primarily conducts a pharmacovigilance analysis of corticosteroids used in ophthalmology by mining voluntarily reported adverse reactions and medication error data from the FAERS database. The classification and standardization of ADRs in FAERS are based on the Medical Dictionary for Regulatory Activities (MedDRA) version 26.1 (Brown et al., 1999). Each report is encoded using preferred terms (PTs), which are categorized into one or more high-level terms (HLT), high-level group terms (HLGT), and system organ class (SOC) levels in MedDRA. The FAERS database is updated quarterly and consists of seven data files: Patient Demographic and Management Information (DEMO), Drug Information (DRUG), Adverse Event Codes (REAC), Patient Outcomes (OUTC), Report Sources (RPSR), Treatment Start and End Dates (THER) for the reported drugs, Drug Administration Indications (INDI), and Deleted Cases. This structured framework allows for standardized reporting and analysis of adverse events related to corticosteroid use in ophthalmology.

### 2.2 Data screening and processing

This study extracted reports from the FAERS database covering the period from the first quarter of 2004 to the fourth quarter of 2023. Given the inclusion of reports for other diseases in direct drug retrieval, we conducted a more refined search using various brand names of corticosteroids commonly used in ophthalmology to minimize false positive results. These brand names included Ozurdex, Maxidex, Dexapar, Dexachlor, Pred Mild, Orchapred, Kapimox-P, Predsol, Trivaris, Triesence, Retisert, Iluvien, Lotemax, Flucon, FML, Visipred, Diflustero, and Durezol (Gaballa et al., 2021). We limited our analysis to reports in which the drug was classified as the primary suspect, using results with the role code ‘PS’. Two researchers were responsible for the data procession by removing duplicates, classifying relevant adverse events through standardized MedDRA queries and PTs, and extracting clinical feature information such as gender, age, reporting region, reporter, route of administration, and dosage form from the reports (Veronin et al., 2020).

### 2.3 ADRs induction time analysis

The induction time of adverse reactions is defined as the interval between the date of the adverse event and the start date of drug use. Adverse reaction induction time was evaluated using quartile tests. In addition, we conducted a Weibull distribution test to assess changes in ADR incidence over time. The scale parameter (α) determines the distribution’s spread, with larger values indicating a wider distribution. The shape parameter (β) affects the curve’s shape; a larger β produces a left-skewed curve, while a smaller β yields a right-skewed curve. Specifically, β < 1 with a 95% confidence interval (CI) less than 1 suggests a decreasing ADR incidence (early failure curve), β close to 1 with the 95% CI including 1 indicates a constant ADR incidence (random failure curve), and β > 1 with the 95% CI not including 1 implies an increasing ADR incidence (wear-out failure curve) (Leroy et al., 2014).

### 2.4 Signal detection and statistical analysis

Disproportionality analysis is a key technique in pharmacovigilance studies for identifying potential safety signals (Montastruc et al., 2011). This study employed various signal detection methods, including Reporting Odds Ratio (ROR), Proportional Reporting Ratio (PRR), Bayesian Confidence Propagation Neural Network (BCPNN), and Polynomial Gamma Poisson Shrinker (MGPS). Among them, ROR is the main method. These methods use a 2 × 2 contingency table to calculate signal strength, as illustrated in Table 1. The study enhances safety signal detection and verification from multiple perspectives by applying these algorithms. This comprehensive approach improves the accuracy of safety signal recognition, minimizes false positives through cross-validation, and enhances the detection of rare adverse events by adjusting thresholds and variances (Michel et al., 2017). Table 2 summarizes the formulas and thresholds for all signal detection methods used. Stronger signals reflect a more significant correlation between drugs and adverse events, aiding in the identification of potential safety issues. All analyses were performed using R 4.3.2 software, GraphPad 10.1.2, and Microsoft Excel for data extraction, analysis, and visualization, ensuring efficient data management and comprehensive analysis. By consulting FDALabel (https://nctrcrs.fda.gov/fdalabel/ui/search), detailed medication instructions can be obtained.

**TABLE 1:** The 2 × 2 contingency table presents the variables used to calculate the reporting odds ratio and proportional reporting ratio.

	Targeted AEs	Other AEs	Total
Reports with ocular corticosteroids	a	b	a + b
Reports with all other drugs	c	d	c + d
Total	a + c	b + d	a + b + c + d

a: Number of reports containing both the target drug and the target adverse drug reaction. b: Number of reports containing other adverse drug reactions related to the target drug. c: Number of reports containing the target adverse drug reaction associated with other drugs. d: Number of reports containing other drugs and other adverse drug reactions.

**TABLE 2:** Principle of dis-proportionality measure and standard of signal detection in this study.

Method	Formula	Threshold
ROR	ROR = acbd=adbc SE (lnROR) = $\sqrt{\left(\frac{1}{a}+\frac{1}{b}+\frac{1}{c}+\frac{1}{d}\right)}$ $95\%cl=\mathrm{e}\ln\left(PRR\right)\pm 1.96\sqrt{\frac{1}{a}+\frac{1}{b}+\frac{1}{c}+\frac{1}{d}}$	a≥3 and 95% Cl (lower limit) > 1
PRR	$PRR=\frac{a∕\left(a+b\right)}{c∕\left(c+d\right)}$ SE ( $\ln\left(PRR\right)$ ) = $\sqrt{\frac{1}{a}-\frac{1}{a+b}+\frac{1}{c}-\frac{1}{c+d}}$ $95\%cl=\mathrm{e}\ln\left(PRR\right)\pm 1.96\sqrt{\frac{1}{a}-\frac{1}{a+b}+\frac{1}{c}-\frac{1}{c+d}}$	a≥3 and 95% Cl (lower limit) > 1
BCPNN	IC = $\log_{2}\frac{P\left(x,y\right)}{P{\left(x\right)}_{P}\left(y\right)}$ = $\log_{2}\frac{a\left(a+b+c+\mathrm{d}\right)}{\left(a+b\right)\left(a+c\right)}$ E (IC) = $\log_{2}\frac{\left(a+\gamma 11\right)\left(a+b+c+d+\alpha \right)\left(a+b+c+d+\beta \right)}{\left(a+b+c+d+\gamma \right)\left(a+b+\alpha_{1}\right)\left(a+c+\beta_{1}\right)}$ V(IC) = $\frac{1}{{\left(\ln2\right)}^{2}}\left\{\left[\frac{\left(a+b+c+d\right)-a+\gamma -\gamma 11}{\left(a+\gamma 11\right)\left(1+a+b+c+d+\gamma \right)}\right]+\left[\frac{\left(a+b+c+\mathrm{d}\right)-\left(a+b\right)+\alpha -\alpha_{1}}{\left(a+b+\alpha_{1}\right)\left(1+a+b+c+d+\alpha \right)}\right]+\left[\frac{\left(a+b+c+\mathrm{d}\right)-\left(a+c\right)+\beta -\beta_{1}}{\left(a+c+\beta_{1}\right)\left(1+a+b+c+d+\beta \right)}\right]\right\}$ $\gamma =\gamma 11\frac{\left(a+b+c+d+\alpha \right)\left(a+b+c+d+\beta \right)}{\left(a+b+\alpha_{1}\right)\left(a+c+\beta_{1}\right)}$ IC-2SD = E (IC)-2 $\sqrt{V\left(IC\right)}$	IC025 > 0
EBGM	EBGM = $\frac{a\left(a+b+c+d\right)\left(a+b+c+d+\beta \right)}{\left(a+c\right)\left(a+b\right)}$ $95\%cl=\mathrm{e}\ln\left(EBGM\right)\pm 1.96\sqrt{\frac{1}{a}+\frac{1}{b}+\frac{1}{c}+\frac{1}{d}}$	EBGM05 > 2

## 3 Results

### 3.1 Basic information of adverse event report

This study obtained safety signals for corticosteroids used in ophthalmology from Q1 2004 to Q4 2023 through data mining. A total of 9,854 ADR reports were retrieved from the FAERS database, including 1,784 reports for Ozurdex, 3,239 for Lotemax, 2,789 for Durezol, 822 for Maxitrol, 455 for Iluvien, 582 for FML, and 193 for Triesence. Other drugs were either not retrieved from the database, had fewer than 50 cases, and were excluded from subsequent analysis.

**FIGURE 2:**
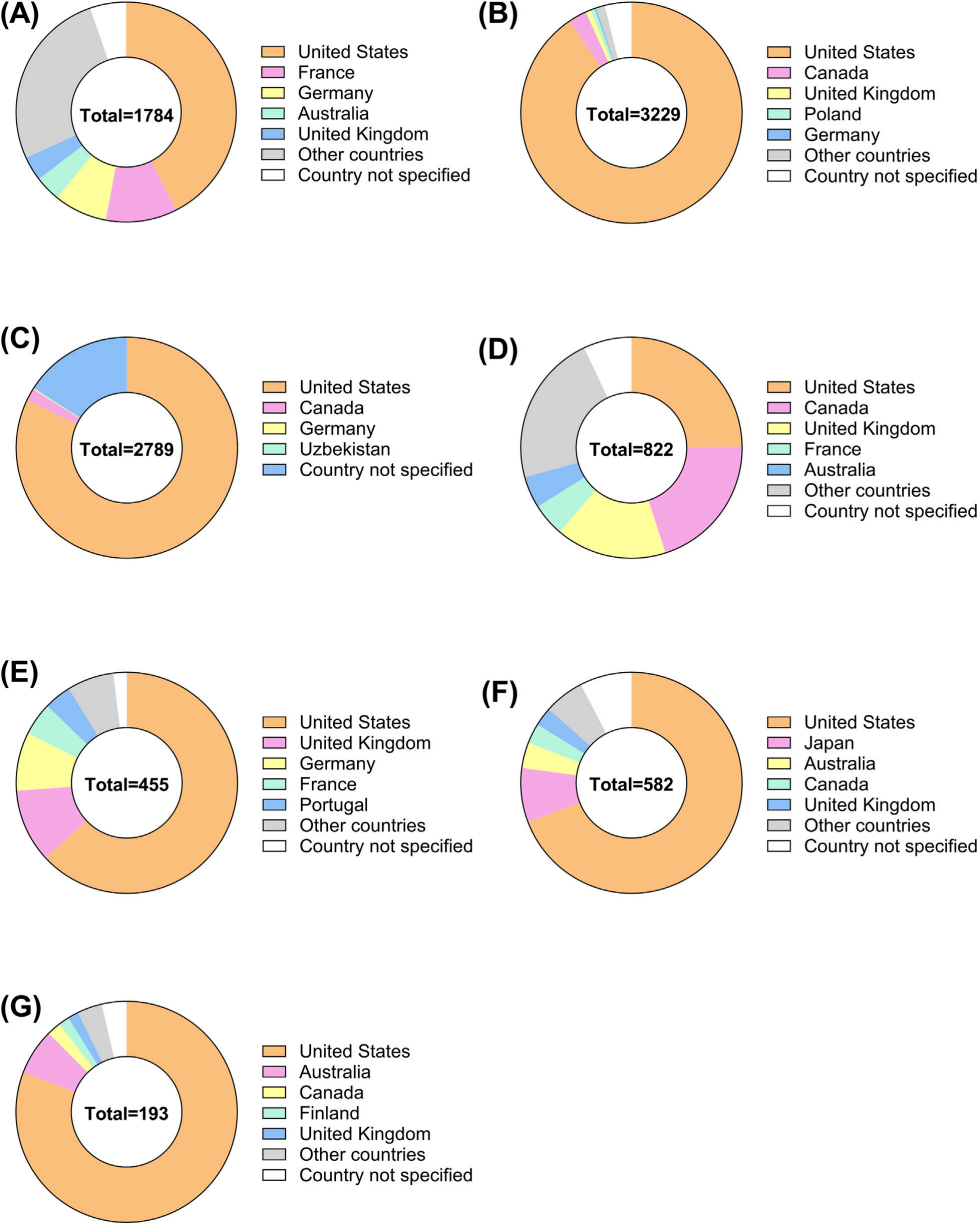
Global distribution of ADRs for ophthalmic corticosteroids by country. The pie charts illustrate the distribution of ADR reports from different countries for ophthalmic corticosteroids, categorized by specific drugs **(A)** Ozurdex, **(B)** Lotemax, **(C)** Durezol, **(D)** Maxitrol, **(E)** Iluvien, **(F)** FML, **(G)** Triesence.

The demographic characteristics of ADR reports related to corticosteroids are summarized in Tables 3, 4, presenting the clinical and report characteristics, respectively. A pie chart was used to visualize the regional sources of these reports (Figure 2), showing that most of the reports were from the United States and European countries. From a gender perspective, except for Ozurdex, women reported 2.3%–49.9% more corticosteroid-related ADRs than men. Most of the reports, excluding those for Triesence, were submitted by healthcare professionals.

**TABLE 3:** Clinical characteristics of patients with ophthalmic corticosteroids-related ADRs.

	Total	Ozurdex	Lotemax	Durezol	Maxidex	Iluvien	FML	Triesence
Corticosteroid		Dexamethasone	Loteprednol etabonate	Difluprednate	Dexamethasone	Fluocinolone acetonide	Fluorometholone	Triamcinolone acetonide
Formula		C_22_H_29_FO_5_	C_24_H_31_ClO_7_	C_27_H_34_F_2_O_7_	C_22_H_29_FO_5_	C_24_H_30_F_2_O_6_	C_22_H_29_FO_4_	C_24_H_31_FO_6_
The marketing authorization date for eye diseases in the U.S.		6/17/2009	09/28/2012	09/30/2008	17/09/2003	26/09/2014	28/07/1982	11/29/2007
Number of individuals	9,854	1784	3,229	2,789	822	455	582	193
Sex								
Female	5,370	645 (36.2%)	2,359 (73.1%)	1,360 (48.8%)	434 (52.8%)	107 (23.5%)	381 (65.5%)	84 (43.5%)
Male	2,875	699 (39.2%)	748 (23.2%)	736 (26.4%)	336 (40.9%)	115 (25.3%)	165 (28.4%)	76 (39.4%)
Unknown	1,609	440 (24.7%)	122 (3.8%)	693 (24.8%)	52 (6.3%)	233 (51.2%)	36 (6.2%)	33 (17.1%)
Weight								
<50 kg	143	5 (0.3%)	65 (2.0%)	20 (0.7%)	32 (3.9%)	0	20 (3.4%)	1 (0.5%)
>100 kg	370	12 (0.7%)	169 (5.2%)	125 (4.5%)	34 (4.1%)	1 (0.2%)	17 (2.9%)	12 (6.2%)
50∼100 kg	1,647	109 (6.1%)	812 (25.1%)	364 (13.1%)	219 (26.6%)	2 (0.4%)	131 (22.5%)	10 (5.2%)
Missing	7,694	1,658 (92.9%)	2,183 (67.6%)	2,280 (81.7%)	537 (65.3%)	452 (99.3%)	414 (71.1%)	170 (88.1%)
Age								
≤17	119	14 (0.8%)	28 (0.9%)	23 (0.8%)	38 (4.6%)	2 (0.4%)	11 (1.9%)	3 (1.6%)
≥86	224	30 (1.7%)	100 (3.1%)	49 (1.8%)	22 (2.7%)	1 (0.2%)	21 (3.6%)	1 (0.5%)
18∼64	2,130	331 (18.6%)	866 (26.8%)	376 (13.5%)	319 (38.8%)	72 (15.8%)	122 (21.0%)	44 (22.8%)
65∼85	2,314	339 (19.0%)	966 (29.9%)	491 (17.6%)	239 (29.1%)	71 (15.6%)	149 (25.6%)	59 (30.6%)
Missing	5,067	1,070 (60.0%)	1,269 (39.3%)	1850 (66.3%)	204 (24.8%)	309 (67.9%)	279 (47.9%)	86 (44.6%)
Outcome of event								
Congenital anomaly	5	0	1 (0.0%)	2 (0.1%)	2 (0.2%)	0	0	0
Death	287	49 (2.5%)	100 (2.8%)	76 (2.5%)	41 (4.2%)	6 (1.2%)	13 (2.0%)	2 (1.0%)
Disability	280	86 (4.4%)	61 (1.7%)	46 (1.5%)	52 (5.3%)	20 (4.0%)	13 (2.0%)	2 (1.0%)
Hospitalization	1,224	194 (9.9%)	405 (11.5%)	288 (9.6%)	197 (20.2%)	33 (6.6%)	92 (14.2%)	15 (7.5%)
Life-threatening	94	5 (0.3%)	22 (0.6%)	18 (0.6%)	34 (3.5%)	0	13 (2.0%)	2 (1.0%)
Others	3,936	1,046 (53.3%)	895 (25.4%)	858 (28.5%)	535 (54.9%)	282 (56.4%)	179 (27.7%)	141 (70.1%)
Required intervention	21	2 (0.1%)	9 (0.3%)	4 (0.1%)	2 (0.2%)	1 (0.2%)	0	3 (1.5%)
Missing	4,978	581 (29.6%)	2036 (57.7%)	1719 (57.1%)	112 (11.5%)	158 (31.6%)	336 (52.0%)	36 (17.9%)

**TABLE 4:** Report characteristics of patients with ophthalmic corticosteroids-related ADRs.

	Total	Ozurdex	Lotemax	Durezol	Maxidex	Iluvien	FML	Triesence
Reporter								
Healthcare professional	5,818	1,251 (70.1%)	1943 (60.2)	1,644 (58.9%)	250 (30.4%)	373 (82.0%)	328 (56.4)	29 (15.0%)
Non-Healthcare professional	3,605	525 (29.4%)	1,084 (33.5%)	1,004 (36%)	527 (64.2%)	74 (16.3%)	230 (39.5%)	161 (83.5%)
Missing	431	8 (0.4%)	202 (6.3%)	141 (5.1%)	45 (5.5%)	8 (1.8%)	24 (4.1%)	3 (1.6%)
Reporting year								
2023	475	162	124	93	39	21	30	6
2022	967	302	274	166	72	97	43	13
2021	951	196	295	233	72	73	77	5
2020	1,086	169	314	320	136	36	94	17
2019	1,212	233	333	346	61	165	51	23
2018	946	139	302	387	40	30	31	17
2017	1,060	131	332	474	53	23	34	13
2016	1,085	107	348	476	52	9	78	15
2015	579	76	262	144	47	1	34	15
2014	360	76	144	53	31	0	25	31
2013	240	74	75	51	31	0	6	3
2012	279	69	116	29	47	0	10	8
2011	130	36	55	9	21	0	9	0
2010	188	14	104	3	35	0	26	6
2009	122	0	64	5	21	0	11	21
2008	62	0	32	0	20	0	10	0
2007	39	0	17	0	19	0	3	0
2006	35	0	21	0	8	0	6	0
2005	22	0	12	0	8	0	2	0
2004	16	0	5	0	9	0	2	0

### 3.2 Analysis of adverse reaction time

**FIGURE 3:**
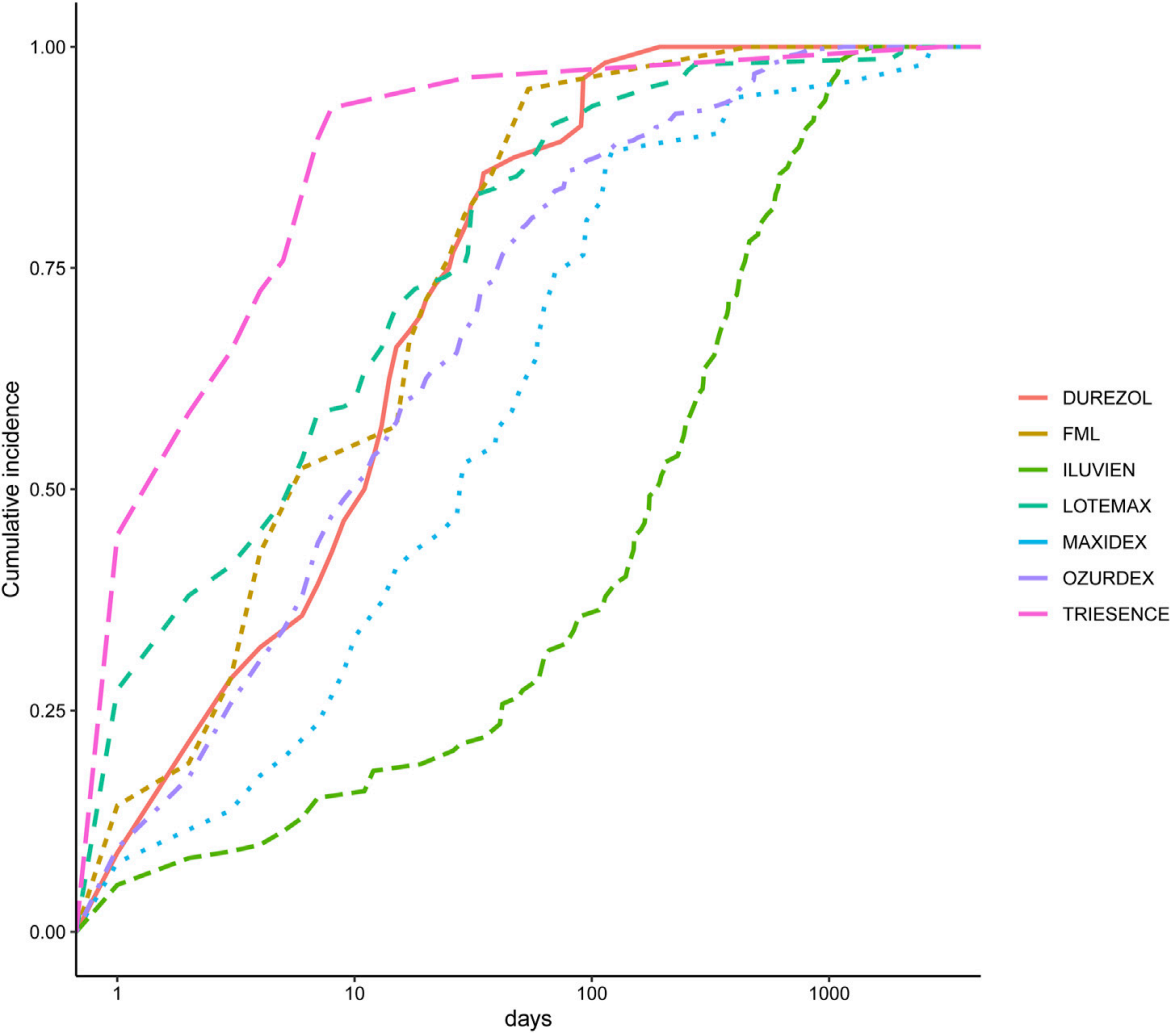
Induction time of adverse reactions for seven different ophthalmic corticosteroids.

We also analyzed the induction time of adverse reactions for these drugs, as shown in Figure 3. The figure shows that the median onset time for adverse reactions is shortest for Triesence and longest for Iluvien. A Weibull distribution test conducted on these drugs revealed that the ADR incidence follows an early failure distribution pattern, indicating a significant decrease in ADR occurrence over time (Table 5).

**TABLE 5:** Weibull distribution test results.

Drug	Median(d) (25%–75%)	Scale parameter: α (95% CI)	Shape parameter: β (95% CI)	Type
Ozurdex	10 (3–40.5)	32.5402454 (24.9826452–40.0978455)	0.5522789 (0.5044848–0.6000731)	Early failure
Lotemax	6 (1–29)	17.8824034 (11.584674–24.1801325)	0.4836633 (0.431619–0.5357077)	Early failure
Durezol	11.5 (3–25.25)	19.5742346 (12.4302627–26.718207)	0.7610364 (0.6133568–0.908716)	Early failure
Maxidex	28 (9–81)	72.0359004 (30.8829917–113.1888091)	0.5106478 (0.4124943–0.6088014)	Early failure
Iluvien	190 (42–442)	254.6536707 (192.1846462–317.1226953)	0.7283638 (0.6250662–0.8316614)	Early failure
FML	6 (3–25)	20.1654305 (4.8068383–35.5240226)	0.5972066 (0.4202388–0.7741744)	Early failure
Triesence	2 (1–5)	7.5668018 (0.05949738–15.074106)	0.3919074 (0.30562079–0.478194)	Early failure

### 3.3 Disproportionality analysis results

**FIGURE 4:**
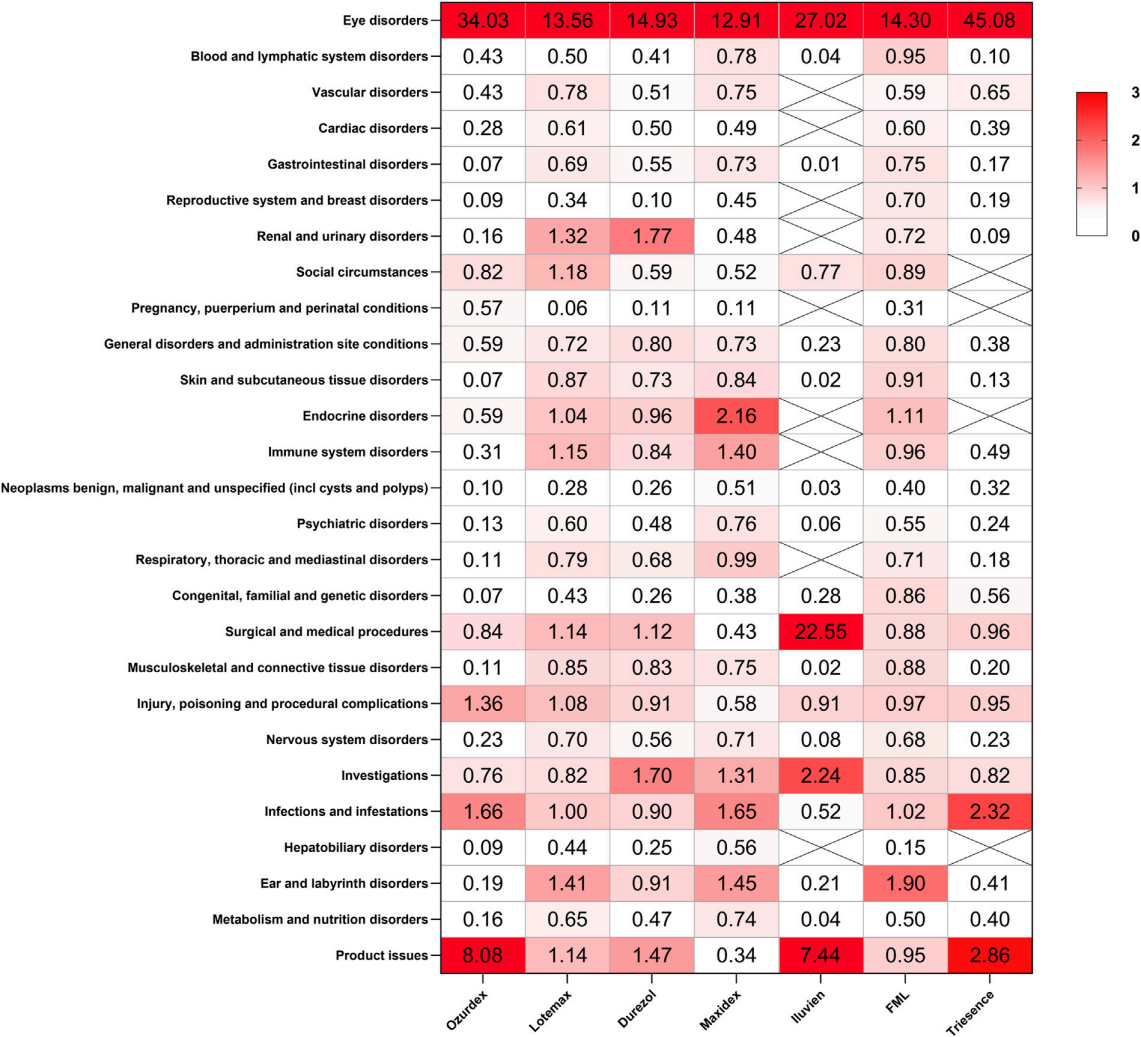
Signal detection analysis of ADRs associated with ophthalmic corticosteroids across various SOCs. The heatmap represents the ROR values for ADRs associated with seven different ophthalmic corticosteroids.

We analyzed the signals at the SOC level for these drugs using the (ROR method, with the results presented in Figure 4. Notable findings include a strong signal for eye disorders across all corticosteroids, with Ozurdex showing the highest ROR of 34.03, indicating a substantial association with ocular ADRs. Additionally, significant signals were detected for renal and urinary disorders with Durezol (ROR = 1.77) and endocrine disorders with Maxidex (ROR = 2.16). Product issues also showed significant signals, particularly for Ozurdex (ROR = 8.08) and Iluvien (ROR = 7.44), suggesting certain risks associated with their intraocular injection procedures. Furthermore, Iluvien exhibited a high signal in the surgical and medical procedures.

**FIGURE 5:**
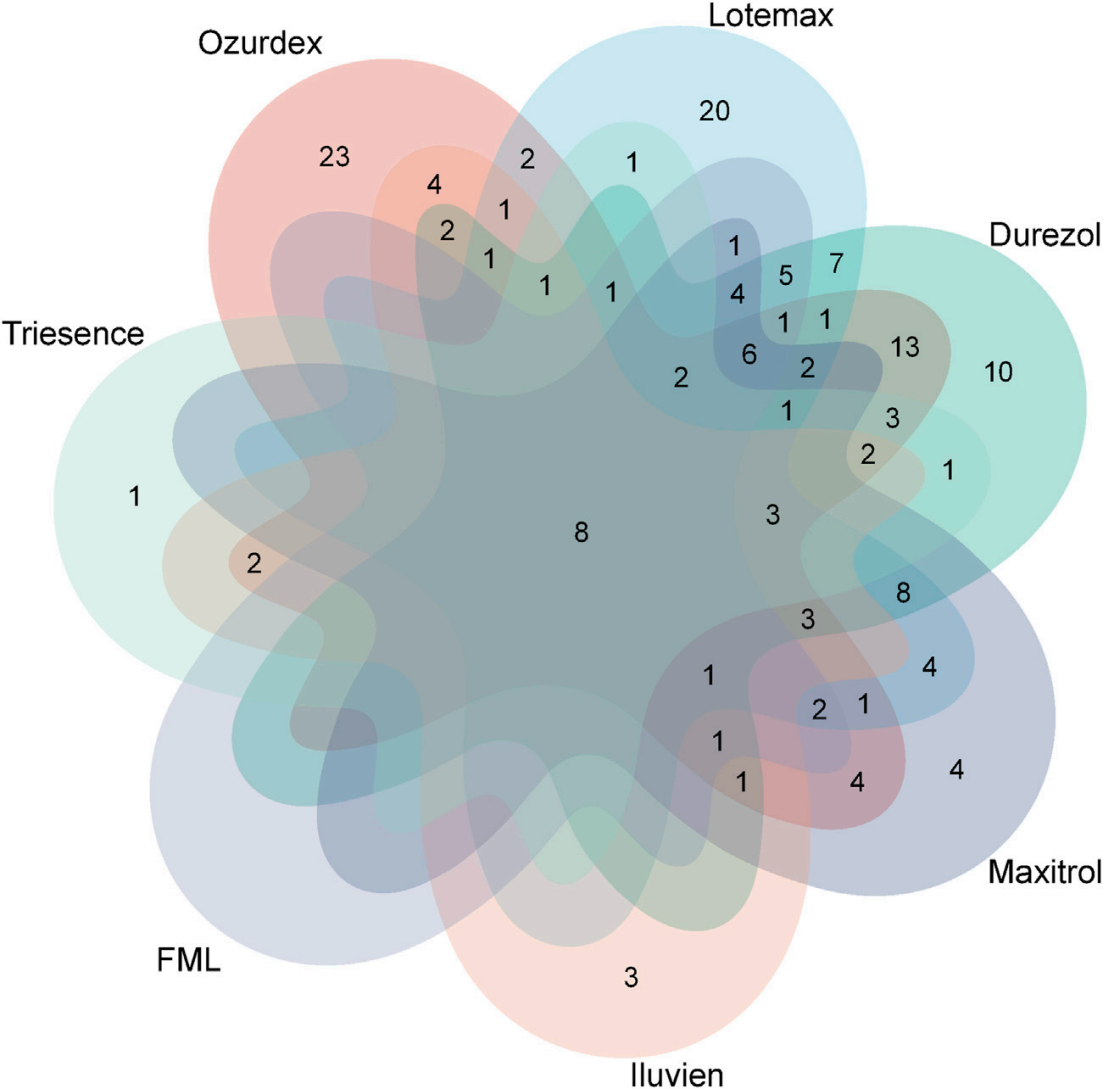
Veen diagram displaying the common ADRs among seven different ophthalmic corticosteroids.

At the PTs level, through screening via four signal detection methods, the number of ophthalmic-related PTs identified were: Ozurdex (92), Lotemax (87), Durezol (88), Maxitrol (56), Iluvien (33), FML (32), and Triesence (26). We used Venn diagrams to visualize the overlap of these PTs across different drugs (Figure 5), revealing a total of seven PTs that appeared in these drugs: eye inflammation, cataract, visual impairment, uveitis, eye pain, vision blurred, visual acuity reduced, and retinal detachment. These shared adverse events highlight common risks associated with these ophthalmic drugs.

## 4 Discussion

Corticosteroids are steroid hormones known for their anti-inflammatory, anti-allergic, anti-shock, and immunosuppressive properties (Barnes, 2006; Kapugi and Cunningham, 2019). Their mechanism of action primarily involves a genomic pathway mediated by glucocorticoid receptors, which bind to specific DNA sites, initiating gene transcription and the synthesis of various proteins (Claman, 1975). These drugs are widely used in ophthalmology, with different molecular structures and dosage forms developed to cater to the specific needs of ocular treatments (McGhee et al., 2002; Gaballa et al., 2021). However, glucocorticoids are also associated with numerous adverse reactions, including increased risk of infections, elevated intraocular pressure, cataract formation, delayed wound healing, and systemic effects such as weight gain, osteoporosis, and hyperglycemia, especially with prolonged use. Clinical practice must carefully manage These potential side effects to balance therapeutic benefits with risks (Carnahan and Goldstein, 2000; Vetro et al., 2022). Pharmacovigilance analysis is an effective tool for monitoring drug safety and identifying potential risks, ensuring that adverse effects are detected and addressed promptly (Ventrice et al., 2013; Gozzo et al., 2021; Vetro et al., 2022). To our knowledge, this study is the first to identify and describe adverse reactions significantly associated with commonly used glucocorticoids in ophthalmology, offering valuable insights into their safety profiles and highlighting the importance of monitoring potential risks in treatment.

In this study, we conducted basic demographic statistics on patients primarily suspected of experiencing adverse reactions related to ophthalmic glucocorticoids, as shown in Table 3. The results showed that the number of women was significantly higher than that of men, which may be due to the higher incidence of most autoimmune diseases in women. Women are generally more frequently affected by these conditions than men (Ngo et al., 2014). Consequently, when using glucocorticoids for treatment, women may require longer treatment durations or higher doses, potentially increasing their risk of side effects. The Weibull distribution test results indicate that seven ophthalmic glucocorticoids follow an early failure distribution pattern, suggesting that ADRs tend to occur in the early stages of drug use, highlighting the need for careful monitoring for adverse reactions soon after initiating treatment.

Maxidex has been linked to endocrine disorders (ROR = 2.16). The associated PT adrenal insufficiency has a count of 13 and a notably high ROR of 19.44, indicating a strong association with the condition studied. Additionally, Cushing’s syndrome has a count of 5 and a high ROR of 18.64, further suggesting a significant association. Similar reports have also been found in many studies, particularly concerning children (Bangsgaard et al., 2018; De Vleeschhauwer et al., 2024). Reports of adrenal suppression with local ocular steroids have also been documented in adults (Sandhu et al., 2012). Given the lack of precise data on the systemic and local concentrations of pediatric eye drops, the use of topical ocular steroids in children requires special attention to avoid potential risks such as adrenal suppression. Durezol was found to be associated with an increased risk of renal and urinary disorders (ROR = 1.77). Through searching related PTs, Chronic kidney disease (CKD) has the highest count (85) and a relatively high ROR of 7.33, indicating a strong association with the condition being studied. End-stage renal disease (ESRD) has a high ROR of 8.62, suggesting that it is also significantly associated, despite having a lower count (24). Renal failure and acute kidney injury have relatively lower RORs (2.84 and 2.09, respectively). Currently, there is no research documenting the relationship between Durezol and renal/urinary diseases. However, corticosteroids are known to directly cause vasoconstriction of arterioles and increase salt and water retention in the kidneys, which may contribute to their impact on renal health (Gibbison et al., 2020). Ozurdex, Iluvien, and Triesence have a high signal regarding product issues, which may be attributed to the invasive procedures involved in administering these medications. Furthermore, Ozurdex and Iluvien, being intravitreal implants with relatively recent marketing authorization, also carry the risk of device dislocation and device malfunction.

A total of seven PTs appeared in these drugs: eye inflammation, cataract, visual impairment, uveitis, eye pain, vision blurred, visual acuity reduced, and retinal detachment. Apart from the clinical manifestations of indications, cataract is the common ADR of these drugs. The increased risk of cataracts is widely acknowledged as a significant side effect of long-term corticosteroid use, although its pathogenesis remains incompletely understood (Smeeth et al., 2003; Wang et al., 2009). Several mechanisms have been proposed to explain this association, including alterations in protein structure and function mediated by glucocorticoid receptors, abnormal cell differentiation influenced by growth factors, modifications in the structure and function of lens proteins, and the role of oxidative stress (Jobling and Augusteyn, 2002; James, 2007). These factors may contribute to lens opacification, ultimately leading to the development of cataracts in individuals undergoing prolonged corticosteroid therapy.

The FAERS database offers significant advantages for studying ADRs. It provides extensive real-world data, enabling the identification of rare or previously unrecognized ADRs, making it an essential tool for post-market drug safety surveillance. By using signal detection methods such as ROR, FAERS data can help screen for potential ADRs and is widely employed in pharmacovigilance efforts. However, the database has its limitations. As a spontaneous reporting system, FAERS data may be influenced by underreporting, misreporting, and duplicate reports, potentially compromising data completeness. Furthermore, FAERS does not support the calculation of ADR incidence rates and lacks detailed patient characteristics and clinical information, which hinders the ability to establish causal relationships. Reporting bias is another concern, as most reports come from Western countries, limiting the generalizability of the findings to other regions. While signal detection can reveal statistical associations, it does not confirm causality between drugs and ADRs. Therefore, FAERS data should be combined with large-scale, well-designed epidemiological studies to investigate further ocular adverse events associated with glucocorticoids in future studies (Gozzo et al., 2023).

## 5 Conclusion

In summary, this study utilized data mining methods to explore the systemic and, particularly, ocular safety concerns associated with currently marketed corticosteroid drugs used in ophthalmology. The findings provide valuable data to support the rational use of corticosteroids in eye treatments and serve as a reference for future safety research. Physicians should remain vigilant for potential adverse reactions, especially in the early stages of glucocorticoid therapy. Early diagnosis and timely intervention can help prevent or mitigate serious consequences stemming from these adverse reactions.

## Data Availability

The original contributions presented in the study are included in the article/supplementary material, further inquiries can be directed to the corresponding author.
